# An Atypical Presentation of Legionnaires’ Disease

**DOI:** 10.7759/cureus.60856

**Published:** 2024-05-22

**Authors:** Hassan Eidy, Barbara Senger, Joshua Steele, Jolian Kathawa

**Affiliations:** 1 Internal Medicine, Corewell Health Hospital, Farmington Hills, USA; 2 Gastroenterology, Corewell Health Hospital, Farmington Hills, USA

**Keywords:** raised liver enzyme, transaminitis, legionella pneumonia, new-onset painless jaundice, legionella pneumophila, extrapulmonary legionella, acute alcoholic hepatitis, legionella infection

## Abstract

Legionnaires’ disease is an atypical pneumonia caused by* Legionella pneumophila. Legionella* species are found in freshwater sources and are transmitted through inhalation of contaminated aerosols. Patients commonly present with fever, chills, and cough. However, in immunosuppressed patients or severe cases, the disease can lead to multiorgan failure. In recent years, the incidence of Legionnaires’ disease has drastically increased and unfortunately is commonly underdiagnosed. Gold-standard diagnosis is made through sputum cultures; however, urine *Legionella* antigen remains the most common test used for diagnosis. Goal-directed care includes antibiotics and supportive care. This case highlights a rare and unique presentation of Legionnaires’ disease presenting with an elevated 2:1 aspartate aminotransferase to alanine transaminase pattern, typically seen with alcoholic hepatitis.

## Introduction

Originally discovered in 1976, Legionnaires’ disease is a subtype of pneumonia caused by the gram-negative bacilli, *Legionella pneumophila*. Patients commonly present with fevers, chills, and either a productive or non-productive cough. Less commonly, patients may present with confusion, nausea, vomiting, diarrhea, and/or anorexia. Diagnosis is usually made through testing the urinary *Legionella* antigen. A chest X-ray may demonstrate basilar consolidation. Laboratory findings may be significant for hyponatremia, elevated erythrocyte sediment rate and C-reactive protein, hypophosphatemia, elevated ferritin, microscopic hematuria, and elevated liver enzymes. Treatment primarily consists of antibiotics with supportive measures [1]. This case highlights a unique and rare presentation of Legionnaires’ disease with a transaminitis trend and physical examination findings mimicking alcoholic hepatitis.

## Case presentation

A 59-year-old female with no known past medical history presented to a satellite emergency department before being transferred to our hospital after five days of fatigue, confusion, and dizziness. She normally resided in California but had been visiting family in Michigan for the last three weeks.

On initial evaluation, she was afebrile and hemodynamically stable. She appeared jaundiced with scleral icterus. She endorsed nausea and anorexia but denied any abdominal pain or emesis. She denied any tobacco or alcohol use and was not taking any prescription medications.

**Figure 1:**
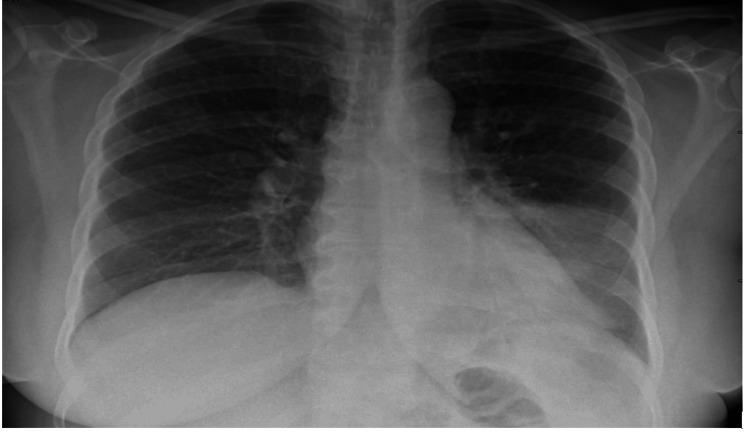
Left lower lobe posterior segmental pneumonia.

Initial laboratory results were significant for transaminitis (aspartate aminotransferase 300 U/L, alanine transaminase 145 U/L, alkaline phosphatase 88 U/L), international normalized ratio 1.2, direct hyperbilirubinemia (total bilirubin 8.1 mg/dL, direct bilirubin 6.6 mg/dL), elevated creatinine kinase (5,750 U/L), neutrophilic leukocytosis (11.9 × 10^9^/L), elevated creatinine (1.31 mg/dL), and hyponatremia (129 mEq/L). Urine drug screen, ethanol, salicylates, acetaminophen, and ammonia levels were negative. Antimitochondrial antibodies, antismooth muscle antibodies, and antinuclear antibodies were also within normal limits to rule out autoimmune etiologies. Human immunodeficiency virus, hepatitis, and infectious mononucleosis testing were unrevealing. Abdominal ultrasound with Doppler was unremarkable. Chest X-ray was suggestive of a left lower lobe consolidation concerning for pneumonia (Figure 1). Urine antigens were checked which were positive for *Legionella* confirming the diagnosis of Legionnaires’ disease.

Initially at the outside facility she presented to she was given a one-time dose of Zosyn 4.5 g. After being transferred to our emergency department and confirming her diagnosis of Legionnaires’ disease, the patient was initially started on intravenous (IV) azithromycin 500 mg receiving one dose before being transitioned to IV Levaquin 500 mg daily for five days. Finally, she was transitioned to oral Levaquin 500 mg daily for two days to complete her full seven-day course before discharge. The patient endorsed symptomatic and clinical improvement throughout her hospitalization with down-trending hepatic function panel and creatine phosphokinase levels (Table 1) before discharge.

**Table 1:** Pertinent labs throughout the hospital course. WBC = white blood cell; AST = aspartate aminotransferase; ALT = alanine transaminase; CK = creatinine kinase

Lab test (units)	Hospital day (HD) 1	HD 2	HD 3	HD 4	HD 5	HD 6	HD 7	HD 8	Reference
WBC (bil/L)	11.6	8.1	9.6	11.6	14.9	13.9	13.6	14.8	3.3–10.7
Sodium (mEq/L)	129	136	137	136	149	138	140	140	135–145
Potassium (mmol/L)	2.5	3.0	3.0	3.1	3.3	2.8	3.2	4.3	3.5–5.2
Phosphorus (mg/dL)	-	-	-	<0.7	1.6	2.3	2.4	2.1	2.3–4.4
Creatinine (mg/dL)	1.24	0.94	0.78	0.77	0.67	0.61	0.64	0.63	0.50–1.10
ALT (U/L)	145	102	94	88	102	95	82	82	8–37
AST (U/L)	300	205	181	192	131	105	112	86	<35
CK (U/L)	5,750	4,986	3,111	1,565	931	460	253	153	30–150

## Discussion

*Legionella* was first isolated as early as 1947 and was confirmed as a distinct causative organism in 1977 by McDade et al. after a severe respiratory outbreak in Philadelphia, Pennsylvania in 1976 [2,3].

According to the Centers for Disease Control and Prevention, cases of Legionnaires’ disease have been on the rise since 2000 with nearly 10,000 cases reported in 2018. However, even with the rise in cases, Legionnaires’ disease remains underdiagnosed and it is possible that the true incidence number is 1.8-2.7 times that of what is actually reported [4].

*Legionella* species thrive in water reservoirs at an optimal temperature of 35°C. Their pathogenesis has been studied extensively over the years. The primary feature of the bacteria is its ability to multiply intracellularly until the cell ruptures and the liberated bacteria infects other cells. *Legionella* enters cells by conventional phagocytosis and resides within a unique phagosome that prevents fusion with lysosomes. It was proposed by Hammer and Swanson that once they enter host cells, *Legionella* causes amino acid depletion and accumulation of 3’,5’-bispyrophosphate. This ultimately leads to an increase in stationary phase factors and genes that facilitate further infection and include sodium sensitivity, cytotoxicity, osmotic resistance, and evasion of apoptosis [5]. Ultimately, this is how *Legionella* species can cause extrathoracic infections spreading to the lymph nodes, brain, kidney, liver, spleen, bone marrow, and myocardium. In 1980, Watts et al. found *Legionella* bacilli in tissue samples obtained from the lung, liver, spleen, and lymph nodes of postmortem patients. This phenomenon distinguishes Legionnaires’ disease from other forms of pneumonia as it can present with elevated serum hepatic transaminases [6]. The patient in this case had hepatic, renal, and muscle involvement and exhibited mental status changes although no indefinite brain involvement was proven.

Currently, urinary antigen testing accounts for 97% of clinical diagnoses of *Legionella*. This test uses monoclonal antibodies that identify most *Legionella pneumophila* serogroup 1 antigens. However, they fail to detect other serogroups of *Legionella pneumophila* as well as other species of *Legionella*. *Legionella pneumophila* serogroup 1 is by far the most common form and accounts for 50-80% of Legionnaires’ disease. Therefore, if urinary antigen testing is the only form of screening, 20-50% of Legionnaires’ disease may potentially remain underdiagnosed. Respiratory culture remains the gold standard for diagnosis as it can identify all known serogroups and species of *Legionella* [7]. In our case, the patient was quickly diagnosed with urinary antigen testing which is by far the most common testing method for *Legionella*.

Legionnaires’ disease has a death rate of 5-10% and will commonly progress during the first week if left untreated [8]. Therefore, it is important to recognize on initial presentation and not allow its unique multiorgan involvement and low sensitivity (85%) of urinary antigen testing to lead clinicians to improper diagnosis. Our patient presented with elevated liver enzymes in a 2:1 aspartate aminotransferase to alanine transaminase pattern, elevated bilirubin levels, and jaundice, a presentation commonly associated with alcoholic hepatitis. Her diagnosis was initially not recognized as *Legionella*. However, with a more thorough history and further workup, she was correctly diagnosed with Legionnaires’ disease and treated accordingly.

## Conclusions

Legionnaires’ disease is an atypical pneumonia caused by infection with *Legionella pneumophila*. If not recognized and treated appropriately, the disease can rapidly progress to cause multiorgan failure with a mortality rate of 5-10% in immunocompetent and up to 30% in immunosuppressed patients. Once diagnosed, it can be treated with various antibiotic regimens with high efficacy. This case highlights the importance of obtaining a thorough history and physical examination in addition to having a broad differential diagnosis.

## References

[1] Brady MF, Awosika AO, Sundareshan V (2023). Legionnaires' Disease. https://www.ncbi.nlm.nih.gov/books/NBK430807/.

[2] Cunha BA (2010). Legionnaires' disease: clinical differentiation from typical and other atypical pneumonias. Infect Dis Clin North Am.

[3] Edelstein PH, Meyer RD (1984). Legionnaires' disease. A review. Chest.

[4] Centers for Disease Control and Prevention (2019). Legionnaires’ Disease Surveillance. Summary Report, United States. https://www.cdc.gov/legionella/health-depts/surv-reporting/2018-19-surv-report-508.pdf.

[5] Fields BS, Benson RF, Besser RE (2002). Legionella and Legionnaires' disease: 25 years of investigation. Clin Microbiol Rev.

[6] Watts JC, Hicklin MD, Thomason BM, Callaway CS, Levine AJ (1980). Fatal pneumonia caused by Legionella pneumophila, serogroup 3: demonstration of the bacilli in extrathoracic organs. Ann Intern Med.

[7] Pierre DM, Baron J, Yu VL, Stout JE (2017). Diagnostic testing for Legionnaires' disease. Ann Clin Microbiol Antimicrob.

[8] (2022). World Health Organization. Legionellosis. https://www.who.int/news-room/fact-sheets/detail/legionellosis.

